# Quality Assessment Across Disciplines in Head and Neck Cancer Treatment Diagnostic Pathology in HNSCC

**DOI:** 10.3389/fonc.2020.00364

**Published:** 2020-03-24

**Authors:** Philip Sloan, Max Robinson

**Affiliations:** ^1^Newcastle EPSRC/MRC Molecular Pathology Node, NuTH and Newcastle University, Newcastle upon Tyne, United Kingdom; ^2^Centre for Oral Health Research, Newcastle University, Newcastle upon Tyne, United Kingdom

**Keywords:** quality assurance, head and neck pathology, molecular pathology, accreditation, cancer

## Abstract

Quality assured pathology services are integral to provision of optimal management for patients with head and neck cancer. Pathology services vary globally and are dependent on resources in terms of both laboratory provision and availability of a highly trained and accredited workforce. Ensuring a high-quality pathology service depends largely on close working and effective communication between the clinical team providing treatment and the pathologists providing laboratory input. Laboratory services should be quality assured by achieving external accreditation, most often by conforming to International Organization for Standardization (ISO) standards such as ISO15189 sometimes with ISO17025 or alternatively ISO17020. Quality of diagnostic reporting can be assured by the ISO but clinical teams should endeavor to work with pathologists who engage in continuing professional development, external quality assurance and audit. Research also contributes to diagnostic reporting quality. A number of initiatives in the UK such as the EPSRC/MRC funded Molecular Pathology Nodes and the National Cancer Research Institute Cellular-Molecular Pathology initiative (C-M Path), for example, have linked pathologists, industry and researchers. This has resulted in centers leading in digital innovation, artificial intelligence, translational research and clinical trials supported by pathologists. For rare tumors and contemporary molecular diagnostics, biopsy material can increasingly be shared with expert specialist pathologists working in specialist centers, particularly by using digital pathology platforms with potentially global reach. High quality services for the majority of diagnostic processes required for head and neck cancer management is best provided by local pathologists where communication with the treating team is more effective than with pathologists working in remote centers. Quality assurance is an increasingly important aspect of pathology, assuring not only effective turnaround times and accuracy for the diagnostic service but also high quality consistent reporting for clinical trials where even small pathology errors can potentially produce a significant bias and in the worst case negate the value of a completed trial. Better outcomes have been associated with centers engaged in clinical trials than in non-participating centers. Provision of a quality assured pathology service should extend to both the research and diagnostic services.

## Introduction

Management of patients with head and neck cancer relies on accurate pathological diagnosis. Quality assurance underpins the pathology service and must cover all stages of the diagnostic pathway from the time tissue samples leave the clinic or operating room to the receipt of the diagnostic report by the clinical team. Interpretation of pathology reports is further quality assured by clinical correlation and discussion at the multidisciplinary team meeting or tumor board. The importance of quality assurance for laboratories globally is recognized by the World Health Organization (1). The WHO Laboratory Quality Management System Handbook sets out international standards and brings together the key documents of the International Organization for Standardization (ISO) and the Clinical and Laboratory Standards Institute (CLSI). The standards set out by CLSI are fully compatible with ISO and it is therefore important for the clinical team to ensure that they work with a laboratory that is accredited by the International Organization for Standardization. Some national laws require accreditation of the whole or parts of pathology laboratory services but in many parts of the world accreditation is voluntary and some diagnostic services lack the resources to achieve accreditation. Pathology accreditation should be to minimum standard ISO15189:2012, though additional accreditation may be offered for specific areas such as Biobanking (ISO 20387:2018) if these activities are undertaken in the laboratory (Table 1). External Quality Assurance (EQA) also plays an important role in driving quality improvement and maintaining a high-quality laboratory test repertoire and the interpretation of those tests by cytologists, pathologists and advanced practitioner biomedical staff.

**Table 1:** International Organization for Standardization and laboratory accreditation.

**ISO standard**	**Description**
15189	Specifies requirements for quality and competence in medical laboratories. Can be used by medical laboratories in developing their quality management systems and assessing their own competence. It can also be used for confirming or recognizing the competence of medical laboratories by laboratory customers, regulating authorities, and accreditation bodies. https://www.iso.org/standard/56115.html
17020	Specifies requirements for the competence of bodies performing inspection and for the impartiality and consistency of their inspection activities. Professional bodies may seek accreditation from ISO under this standard and then use their own guidelines for laboratory accreditation. https://www.iso.org/standard/52994.html
17025	Specifies the general requirements for the competence, impartiality and consistent operation of laboratories. It is applicable to all organizations performing laboratory activities, regardless of the number of personnel. Laboratory customers, regulatory authorities, organizations and schemes using peer-assessment, accreditation bodies, and others use this standard in confirming or recognizing the competence of laboratories. https://www.iso.org/standard/66912.html
20387	Specifies general requirements for the competence, impartiality and consistent operation of biobanks including quality control requirements to ensure biological material and data collections of appropriate quality. This document is applicable to all organizations performing biobanking, including biobanking of biological material from multicellular organisms (e.g., human, animal, fungus, and plant) and microorganisms for research and development. Biobank users, regulatory authorities, organizations and schemes using peer-assessment, accreditation bodies, and others can also use this document in confirming or recognizing the competence of biobanks. For entities handling human materials procured and used for diagnostic and treatment purposes ISO 15189 and other clinical standards are intended to apply first and foremost. https://www.iso.org/standard/67888.html

**Figure 1:**
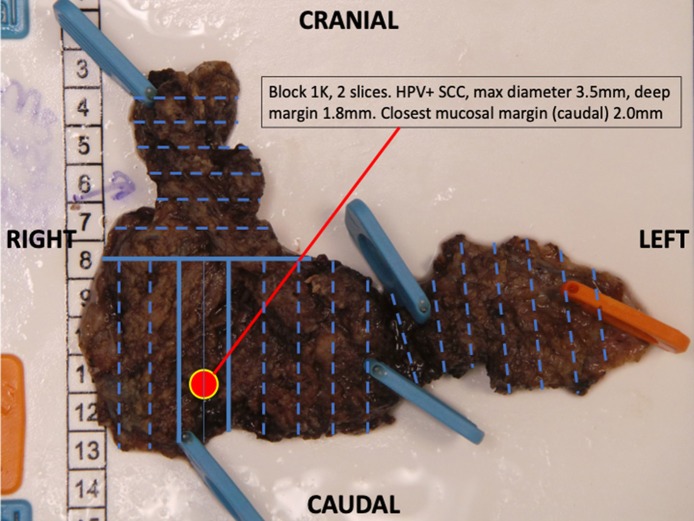
A transoral robotic excision of base of tongue to identify an unknown primary lesion. Following the agreed protocol, the resection has been pinned mucosa side down. The entire specimen is blocked out (Blue lines) and annotated so that the primary lesion can be located to aid further management.

Proper documentation is essential to provision of a high quality diagnostic service. Standard Operating Procedures (SOPs) are used to document a series of detailed protocols and working procedures that can be followed by all of the laboratory staff so that a continuous quality service can be provided. SOPs must be regularly updated and held in a central repository so that only current documents are used for service provision. It is important to involve the whole clinical head and neck team in the preparation of those SOPs that relate to clinical practice and communication. The use of transoral robotic surgery (TORS), for example, has necessitated the formulation of new pathology protocols for handling the surgical specimen. The protocol can best be optimized through discussion between the surgeon and pathologist (Figure 1). Good laboratory services seek to continuously improve and implement innovations and should welcome regular external inspections and regulatory visits to maintain quality. Effective communication between the pathologists and clinical team is vital and there should be SOPs to cover communication. There are risks associated with multiple pathology reports and separate ancillary and molecular test reports. Laboratory information management systems (LIMS) should aim to collate this information into integrated pathology reports which ideally would automatically upload into a comprehensive electronic medical record. The multi-disciplinary team meeting (MDTM) or tumor board should include pathologists as core members to facilitate effective communication and service improvements.

## Quality Assurance in the Laboratory

Almost all head and pathology is undertaken within large multi-disciplinary laboratories that have documented quality assurance procedures in place. It is not practical for very small laboratories to obtain accreditation and many have merged with larger laboratory services. A quality manager is essential to ensure that all processes and procedures are being correctly carried out in the laboratory. Accreditation in the pathology laboratory is generally to minimum standard ISO 15189: 2012. In the UK and Ireland accreditation to ISO 15189 is mandatory and regulated by the United Kingdom Accreditation Service (UKAS). Increasingly ISO 15189 accreditation is required by countries in continental Europe and the standard is being rolled out globally. Other standards may be applied for example in Finland and Switzerland accreditation to ISO 15189 and ISO 17025 is required covering both the clinical decision making and metrical aspects. In Germany, accreditation to ISO 15189 is voluntary for a pathology laboratory. A minimum requirement, however, is compliance with the Quality Assurance of Medical Laboratory Testing Guideline (Rili-BÄK) issued by the German Medical Association, and this standard is accredited under ISO 17020. There is a bias in ISO 15189 toward laboratory processes whereas the Rili-BÄK guideline covers the whole diagnostic service including both the laboratory processes and reporting standards of the pathologists (2). Accreditation organizations in the USA must submit proof that their practice standards meet the minimum requirements set out by the Clinical Laboratory Improvement Amendments (CLIA) regulations (3).

According to the ISO accreditation guidelines, all processes, procedures and examinations related to pathology diagnostics must be documented as standard operating procedures (SOPs) that are current and accessible to the laboratory staff. Initial documentation of these processes, procedures and examinations allows the laboratory head, manager and staff to perform internal evaluations that can eliminate unnecessary steps and improve efficiency and accuracy. These collected records (SOPs and their precursor “working instructions”) comprise an enduring intellectual property of the lab, guaranteeing that experientially gained technical knowledge will be maintained without regard to personnel changes. Finally, they create a basis for a standardized rather than experiential induction for new employees into the work process.

Head and neck pathologists working within large multidisciplinary laboratory services participate in laboratory accreditation through creation and updating of SOPs and also by audit (see below). It should be remembered that accreditation to ISO 15189 relates to diagnostic procedures and processes within the laboratory but does not assure overall diagnostic quality. Head and neck pathologists should participate in quality assurance, audit, and educational events to ensure ongoing competency in diagnostic reporting and clinical trials if included in their practice.

Competency assessment for all staff is an essential part of quality assurance in the pathology laboratory. Responsibility for provision of a quality diagnostic service reaches out beyond the laboratory itself to a whole range of staff including those who transport and receive samples, medial secretaries who handle patient data, IT support, biomedical staff, managers, and advanced practitioners who are authorized to issue reports. It is of key importance that any person in the laboratory performing a task is competent to undertake that task. Such competencies must be documented and should form part of an activity log for laboratory staff who are on an approved training pathway programme (2).

## Quality Assurance for Head and Neck Pathologists

### Training

**Figure 2:**
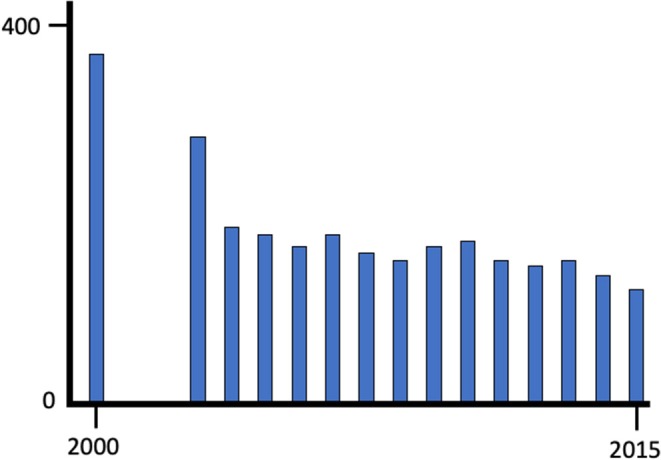
The decline in clinical academic posts in pathology between 2000 and 2015 in the United Kingdom; data from the C-M Path website (accessed 05.05.2019). Over the same period clinical academic posts as a whole remained relative stable.

In order to develop the skills and knowledge to provide a high-quality pathology service, it is important that trainees have the opportunity to engage in a properly structured programme delivered by pathologists who are motivated to provide high quality education. Recruitment is a key factor and some programmes have not been able to attract sufficient numbers of high quality trainee applicants to maintain the workforce. In the UK for example there has been a steady decline in the numbers of academic pathologists over the last 15 years (Figure 2). A similar trend of declining workforce has been recognized in North America where from 2010 to 2019 over 40% fewer US medical students chose to pursue pathology residency programmes (4). The reasons for difficulty in recruitment to pathology are uncertain and several factors have been cited. Revision of medical curriculum in many medical schools has resulted in less undergraduate exposure to pathology. Pressures on the pathology service have resulted in pathologists having less time to provide teaching and the falling numbers of academic pathologists has compounded this in the UK and elsewhere. Remuneration and reward for junior doctors may also affect recruitment if trainee pathologists are disadvantaged compared to other specialities.

An important aspect of training is programme structure and competency assurance. In the United Kingdom for example, the Royal College of Pathologists sets out the curriculum and provides examinations that assure competency as the trainee progresses through the training stages. The College of American Pathologists has a similar function in the United States where residency programmes lead to Board certification. In some areas of pathology such as neuropathology and forensic pathology, sub-speciality training must be followed. However, head and neck pathology is not generally recognized as a sub-speciality for training. Pathologists who wish to practice in head and neck pathology follow the general pathology training route and gain specialist training post-qualification by engaging in specialist practice with experienced colleagues or through courses and research. Globally, training is more variable and often the pathology department itself has a greater degree of autonomy in determining curriculum, examinations and competency assurance. Training records are helpful for self-assessment of progress and are essential particularly if pathologists wish to relocate in the future. As with other disciplines, training in pathology is a mixture of academic knowledge and skill sets documented by meetings records, competency assessments and examination results with clinical practice. It is important for trainees to document their clinical activity and experience throughout the programme, including specimen numbers and types as well as complex trimming and autopsy experience. In that way, a comprehensive record of training can be built that may be used to provide evidence of satisfactory training. Workplace based assessments such as directly observed procedures and extended case based discussions should be regularly undertaken and can be assessed at an annual review of competency progression. Independent practice is very important as the trainee progresses. Audit of trainee reports by senior pathologists provides both quality assurance for the clinic and useful feedback for developing competency. Training programmes vary internationally but should set out a clear curriculum, objectives, experiential requirements and competency assessment processes, with a certificated outcome. Most substantive pathology posts are currently advertised with a requirement for one or more specialist areas and increasingly pathology departments are organized into specialist teams able to mentor newly qualified pathologists. Many pathologists who specialize in head and neck pathology practice in an additional complementary specialist area such as dermatopathology, endocrine pathology or bone, and soft tissue pathology.

Interestingly, more than one pathway of training for head and neck pathology exists in several countries. Oral and maxillofacial pathology training pathways are open to dentally qualified individuals. Quality assurance requirements and health economic considerations have resulted in merger of small dedicated oral and maxillofacial pathology laboratories into larger centralized laboratory services. Oral and maxillofacial pathologists working in such a setting have typically expanded their range of practice and undertake head and neck work and often a second speciality. Alternatively, medically qualified trainees may follow a conventional general pathology pathway and then specialize in head and neck pathology, often in combination with another speciality. As skill mix changes are developed it is likely that many tasks currently performed by pathologists such as dissection of surgical specimens and reporting of less complex cases will be undertaken by non-medically qualified advanced practitioners working within the head and neck pathology team.

### External Quality Assurance Schemes

**Figure 3:**
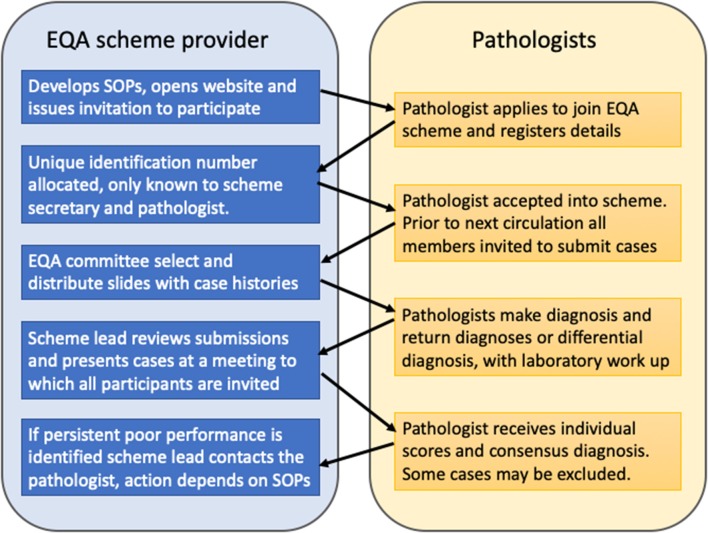
The United Kingdom external quality assurance scheme cycle. Cases are contributed by scheme members and bi-annual slide review ensures quality, through feedback, and discussion. Cases where no consensus diagnosis can be reached are designated “educational” and excluded from the scoring scheme.

Pathologists must be up to date with recent developments in the field including new entities, tumor classification and increasingly molecular pathology testing for diagnosis and targeted therapies. It is also important for pathologists to assure themselves that their competencies have been maintained. Participation in external quality assurance (EQA) schemes ensures quality and forms an important part of continuing professional development (CPD, see next section). Pathology practice is increasingly specialized and there are now several specialist EQA schemes as well as general schemes in the United Kingdom. The principle is essentially the same for both types of scheme (5). Diagnostic slides are contributed to the EQA co-ordinator who makes up sets of slides and distributes them with case histories to the participating centers (Figure 3). Pathologists then make their individual diagnoses, or if a differential diagnosis is appropriate give their preferred diagnosis in ranked order and state how they would reach a definitive diagnosis. Participants are given a number known only to themselves and the scheme manager ensuring anonymity. There is typically circulation of a new slide set every 6 months. For head and pathology there may be subsections of oral and maxillofacial pathology, ENT pathology and common slides, with participants able to undertake selected sections or the whole set. Once the returns are made a national meeting is held (often as a satellite of a specialist society meeting) to which all participants are invited. A consensus diagnosis is reached at the meeting based largely on the returns but also through discussion. If no consensus can be reached then the case is declared educational and excluded from the marking scheme. Pathologists participating in the EQA scheme later receive their individual mark along with information about the submitters diagnosis, the consensus diagnosis, and results of any molecular testing not previously given in the history. Statistical data relating to the overall marking is also provided so the individual pathologist can measure their own performance against that of the participating group as a whole. A reflective note can be written for cases out of consensus that forms part of the CPD record. Where there is significant underperformance, for example benign disease confused with cancer, then the EQA scheme organizer may contact the pathologist through the manager. Typically, underperformance in any particular circulation is usually followed by improvement in the next round. When a pattern of persistent underperformance is found, then the scheme organizer will contact the individual and ascertain the reasons. Ultimately, in the UK, the Royal College of Pathologists may be notified and the medical director of the hospital can also be informed and local investigation may take place. Fortunately, persistent underperformance is very rare.

Head and neck pathology services are provided by oral pathologists, specialist head and neck pathologists and general pathologists. To assure quality, it is desirable for these pathologists to enroll in an interpretive external quality assurance scheme, such as that provided by the BSOMP (https://www.bsomp.org.uk/eqa) accredited by the Royal College of Pathologists. The scheme has many members outside the UK and participation can be done using digital pathology with archived slide sets available for reference (https://www.virtualpathology.leeds.ac.uk/eqa/specialist/headneck). Unfortunately, EQA schemes in head and neck pathology are rare outside of the UK, even though analysis of the UK scheme showed wide participation and encouraged oral and maxillofacial pathologists to broaden their practice and improved quality (5).

### Continuing Professional Development

An important part of quality assurance for head and neck pathologists is their participation in relevant meetings and educational activities, including on-line modules and self-assessment packages. Such activities must be documented and considered as part of an appraisal or maintenance of competency recording. In North America, the Canadian Association of Pathologists- Association canadienne des pathologistes provides on-line modules including head and neck pathology. The Royal College of Pathologists hosts a CPD scheme open globally through affiliate membership. In Europe, the European Society of Pathology accredits meetings for continuing medical education (CME) points that include head and neck pathology. Similar schemes exist in many countries and participation is essential to maintain competency and implement new knowledge and practices into the service. From a global perspective, quality assurance can help developing pathology services in countries where resources are limited. Services are advancing rapidly and fostering international communication and training opportunities is an essential part of achieving worldwide high standards. Setting of international standards such as defining the classification and genetics of head and neck pathology (6) and producing accessible guidelines for minimum datasets (7) can form the basis for self-assessment and define areas where CPD can be useful. The work of international committees such as those of medical charities and the Royal College of Pathologists (https://www.rcpath.org/international/about-international.html) can also drive quality assurance through provision of training opportunities and CPD.

### Case Consensus Meetings

Increasingly, head and neck pathology is provided by specialist pathologists who work in small teams often in centers providing head and neck oncology services. An important part of quality assurance on a day to day basis is the “double reporting” of cancer cases either in real time or at a dedicated weekly meeting around a multi-headed microscope. Through holding a regular consensus meeting, colleagues can not only look at cancer histology slides and discuss interpretation, but can also build a local database of cases. The consensus meeting also affords the opportunity to discuss implementation of new practices and monitor laboratory quality issues. A short weekly meeting is more effective than a programme of less frequent lengthy meetings, as issues can be resolved quickly. In head and neck pathology, it is often useful to discuss cases where agreement is known to be poor between pathologists, such as the presence or absence of extra-nodal extension in metastatic deposits, grading of epithelial dysplasia, interpretation of small poorly orientated biopsies, equivocal immunohistochemistry, HPV status and rare disorders. The consensus meeting is also useful for the education of trainee pathologists. A Standard Operating Procedure should cover the recording of the consensus meeting data as this forms part of the hospital record. Telepathology can be used to link pathologists working in accredited pathology services to colleagues in low resource areas to help reach a consensus diagnosis as well as facilitating external quality assurance schemes and post-graduate training (8).

### Audit

**Figure 4:**
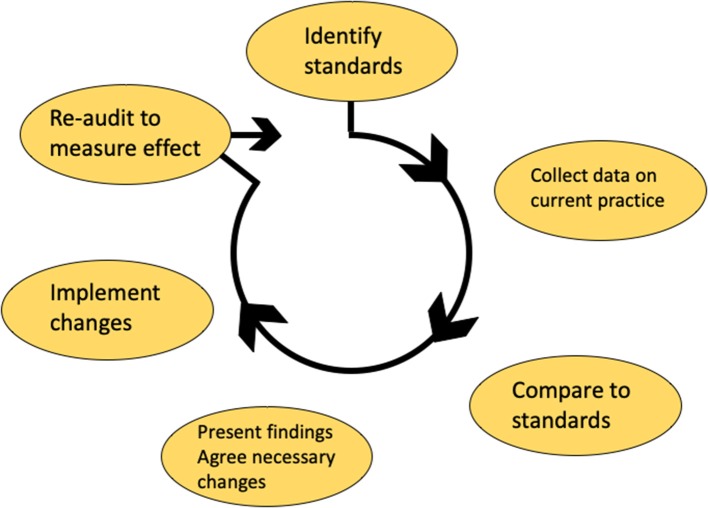
The audit cycle used in the United Kingdom NHS service. Standards are first identified and the process to be audited is measured against the gold standard. Once changes identified by the audit are implemented, then the process should be re-audited until standards are met.

Clinical audit is a process that seeks to identify where improvements can be made within healthcare services by measuring them against evidence based standards. Specific areas for quality improvement can then be targeted to ensure that patients receive the best possible care. In order to maintain safe and high quality practice in head and neck pathology, it is important to audit the service. Individuals and teams can then demonstrate that their practice and procedures meet standards. Clinical audit is the best method for generating this evidence. Audit topics may be identified by local issues or patients' concerns, hospital, and laboratory priorities, new guidelines, treatments of procedures and cost-effectiveness. A specific aim should be identified that measures a gap between ideal practice (determined from evidence, guidelines and standards) and actual practice. Appropriate standards to compare practice against must be identified and where possible published international, national, regional, or local standards should be selected. If published standards do not exist then objectives can be developed and research evidence, past audits and consensus opinion can be used to formulate a gold standard. Once the audit has been completed a report or presentation should be prepared and change can be implemented. After a suitable period of time a re-audit should take place to judge whether changes have been effective, thus completing the audit cycle (Figure 4). Audits can be submitted for formal evaluation by peer review at the Royal College of Pathologists in the UK who also provide open access guidance on the principles of conducting a high-quality clinical audit (www.rcpath.org).

### Patient Safety Systems

Patient safety companies such as Datix (Swan court, London) and Global Research for Safety (Gesellschaft für Anlagen- und Reaktorsicherheit, Cologne) offer software packages that aim to capture clinical incidents and thus enable risk reduction by learning from errors that have occurred and thereby enhance quality assurance. Clinical errors that occur in head and neck pathology can be logged in such databases if implemented in the hospital management system. Reporting of incidents enables efficient identification of areas for improvement and training that may be required. This helps not only to create a patient safety culture and also to mitigate future risks. The advantage of participation for head and neck pathology is that incidents can be viewed in the wider clinical context, providing insights for pathologists and the clinical team. Errors that occur in the pathology laboratory may ultimately lead to a clinical incident and it is important that feedback is provided to the laboratory quality manager so that changes can be implemented with urgency if sub-optimal practice is identified. It is good practice for quality managers to maintain a “dashboard” to monitor critical incidents and “at risk” activities, for example when staff absence, reagent supply or machine failure threatens the service. Rapid communication with the head and neck clinical team is vital to ensure patient safety in case of discovery of an error and withdrawal of a laboratory reagent because of failure uncovered by quality control. Patients affected must be quickly identified and appropriate remedial action taken.

## Quality Assurance for Digital Pathology

There is huge interest globally in digital pathology for provision of routine clinical reporting, education and quality assurance. Digital pathology can be integrated with other digital tools such as barcoding, specimen tracking and voice recognition to ensure a seamless cellular pathology workflow. Such systems also enable effective archiving and offer the possibility to link many types of clinical data. Digital pathology has the potential to bring about improvements in the safety, quality and efficiency of a cellular pathology department. Much has been written about the possibility of using artificial intelligence (AI) systems and it is likely that many innovations will be implemented into head and neck pathology in the future whilst others may not be validated for clinical use.

As with any rapidly advancing technology, it is important that validation takes place at every stage of implementation of digital pathology. Further, the regulatory framework must be complied with to ensure proper accreditation of the pathology service. Slide scanners and image analysis algorithms when intended for medical use (including diagnosis) are classed as medical devices (9, 10). The US FDA is testing a new Pre-Cert model (10) with the intention of demonstrating by premarket review and excellence appraisal that the same quality of information as a traditional approach to ensure safety and effectiveness standards are met. Pre-market review of digital health tools as medical devices includes implementing a new approach to the review of artificial intelligence tools. Formal studies of digital vs. conventional slide based assessment are required. Recently for example it has been shown that a group of pathologists could achieve 100% concordance on reporting of immunohistochemistry (11). However, it was found necessary to scan slides at x40 resolution rather than x20 to achieve confident digital reporting. This level of detail is necessary in order to develop detailed protocols and SOPs for routine practice. In head and neck pathology interpretation of *in situ* hybridization for high risk HPV DNA can be challenging and may require careful study of glass slides using high magnification at different focussing planes. It is not known whether digital pathology could be used for such an application and validation would be needed before implementation into the diagnostic service. Another example is that research has demonstrated that tumor infiltrating lymphocytes (TILs) in head and neck squamous cell carcinoma are highly prognostic and can sub-stratify HPV associated oro-pharyngeal carcinoma (12, 13). Algorithms have been developed that can measure TILs but using such data to provide make clinical decisions must be viewed with caution until AI testing has been validated and accredited for clinical use. Equally, it is likely that testing based on AI algorithms will underpin future targeted or immunomodulatory therapies for head and neck cancer (14).

## Quality Assurance for Clinical Trials

Accurate pathological diagnosis is central to clinical trial entry and treatment stratification. Molecular testing often divides traditional entities into smaller subcategories requiring large multicentre, often multinational, interventional studies. Such studies require quality assured laboratory services, high diagnostic standards and validated reporting uniformity. Central pathology case review was first adopted in the 1960s following the identification of poor inter-observer variation between pathologists assessing lymphoma as a source of bias (15). Widespread central pathology case review occurs in clinical trials and is particularly valuable where rare or morphologically challenging diagnostic disorders are being considered. Currently, most central reviews occur after implementing patient management decisions for quality control prior to publication, rather than in “real time” for trial entry. One example is the central review of sentinel nodes in the SENT trial where surgical centers contributed slide sets for review by a group of trial pathologists (16). Only two discrepancies were identified; both where the local pathologist had reported individual tumor cells that were considered to be cytokeratin positive non-viable cell debris by the trial pathology group. Both patients had undergone neck dissection with no tumor found and were excluded from the analysis. The central review process led to greater understanding of interpretation of sentinel nodes in the context of metastatic oral cancer and formulation of guidelines for pathology. Central review that involves reviewing slides risks loss or breakage during transportation and it may not be possible to produce replacement slides from limited remaining tissue. Digital pathology has the potential to ameliorate many of these issues. Scanning of trial slides and image storage should be considered when planning new clinical trials where pathology is involved. Shortage of skilled trial pathologists is becoming a key issue in the conduct of clinical trials within the UK (17, 18). Digital pathology enables linking of distant pathology centers and could expand access to expert pathologists. Real time dissemination of identical images to multiple centers can allow simultaneous case review, reducing turnaround times and ensuring consensus opinion before therapeutic allocation (19). Diagnostic re-classification at the end of a study may identify suboptimal patient care and negate the significance of investigational findings. Even minor errors in diagnostic accuracy can affect the statistical significance of trial outcomes (19, 20). In head and neck pathology, real time central HPV testing has been implemented for clinical trials recruiting patients with oropharyngeal cancer (e.g., DeEscalate HPV and PATHOS), the latter also includes quality assurance of the surgical pathology (primary resection and neck dissections) to ensure patients are allocated to the correct risk group in the trial protocol (21, 22).

## Head and Neck Guidelines and Standards

### International Collaboration on Cancer Reporting

In order to quality assure any head and neck service, it is important that pathological data are recorded in a consistent way. The International Collaboration on Cancer Reporting (ICCR) aimed to define a portfolio of minimum datasets available globally (7). Nine new datasets for head and neck pathology were published in September 2018. Each minimum dataset identifies elements that are mandatory and advisory. An accompanying paper has been published for many datasets and there is always useful narrative that accompanies each dataset, providing guidance on interpretation and rationale of the dataset. The ICCR datasets harmonize the previous datasets provided by Colleges and professional associations around the world and many of these organizations have endorsed the datasets. Pathology departments in hospitals treating head and neck cancer may simply check that their current recording systems are compliant with ICCR, or they may decide to incorporate the ICCR proformas into their reporting system. Minimum datasets are currently available for:

- Carcinomas of the Oral Cavity
- Carcinomas of the Hypopharynx, Larynx, and Trachea
- Carcinomas of the Nasopharynx and Oropharynx
- Carcinomas of the Major Salivary Glands
- Carcinomas of the Nasal Cavity and Paranasal Sinuses
- Ear and Temporal Bone Tumors
- Malignant Odontogenic Tumors
- Mucosal Melanomas of the Head and Neck
- Nodal Excisions and Neck Dissection Specimens for Head and Neck Tumors.

### Staging and Diagnostic Entities

In order to quality assure head and neck outcomes it is important that accurate staging data are recorded. The AJCC and UICC TNM8 provide up to date guidance and is used in the ICCR datasets. For consistency, the published UICC text and any electronic versions must be updated to correct errors using the published errata [https://www.uicc.org/sites/main/files/atoms/files/UICC%20TNM%208th%20Edition%20Errata_09.05.2017.pdf]. For the first time in head and pathology, TNM8 has separate categories for clinical and pathological staging. Use of a biomarker (p16) to identify HPV associated oropharyngeal cancer is now mandatory as different staging is used for HPV positive and negative cases. The WHO Pathology and Genetics series provides a global standard for definition of diagnostic entities and the head and neck volume was last updated in 2017 (6). For quality assurance, it is important for pathologists to use this series as a reference standard. As evidence accumulates, then the series is updated. Other pathology literature such as authoritative textbooks and original scientific articles should also be used to provide evidence for good pathology reporting practice. Guidelines for pathology in head and neck are provided by the College of American Pathologists (CAP), National Comprehensive Cancer Network (NCCN), American Society of Clinical Oncology (ASCO) and UK Multidisciplinary Guidelines for Head and Neck Cancer. With a multiplicity of guidelines in the literature, harmonization should be aimed for wherever possible and evidence cited in a reference section that reflects source data. Local guidelines always have to be agreed to match services with resources available. It is not always possible for every treatment center to follow every aspect of international guidelines. Patients should be informed and local guidelines followed by the treatment center.

## Quality Assurance for Molecular Testing in Head and Neck Pathology

Whole genome sequencing or whole exome sequencing is being introduced into clinical service, though at present most molecular testing utilizes validated immunohistochemistry, cytogenetic methods or panel sequencing. In the UK, whole genome sequencing is being introduced into the NHS clinical service from July 2019 though only for sarcoma, hematological malignancy and pediatric oncology initially. Whole genome sequencing can be of value in head and neck squamous cell carcinoma, where prognostic subsets can be identified that may ultimately guide therapy (23–26). In the pathology laboratory cellularity scoring is necessary to ensure that sufficient tumor DNA is present in a sample; currently for whole genome sequencing 40% tumor cells from has been set as a threshold with DNA quality control after extraction from 5 mm cube of fresh tissue. It is likely that the tumor volume and threshold of cellularity will be reduced as technology develops. The results of the UK external quality assurance scheme have shown that there is wide variation in cellularity scoring amongst pathologists. This has prompted the production of an open access on line training package for pathologists (https://www.genomicseducation.hee.nhs.uk/courses/) that covers the principles and pitfalls of cellularity scoring on sections. These relate to issues around 3-dimensional architecture and the relative size of cell nuclei, both of which tend to lead to overestimation of the ratio of the genomes, particularly in lymphocyte rich tumors.

Quality assurance for biomarkers such as PDL-1 can be provided through training packages and evaluation (https://www.agilent.com/en/product/pharmdx/pd-l1-ihc-28-8-pharmdx-interpretation-training). Quality assurance schemes for immunohistochemistry are provided by NordiQC (http://www.nordiqc.org/) and NEQAS (https://www.ukneqasiccish.org/). The College of American Pathologists accredits laboratories and advises on quality assurance for immunohistochemistry, based on defined principles (27). Clinical trial pathologists should undertake Good Clinical Practice (GCP) and Good Clinical Laboratory Practice (GCLP) training with documented refreshment of learning biannually. These set out internationally recognized basic standards and are a minimum requirement for non-accredited laboratories involved in biomarker research and application. Both GCP and GCLP are broad ranging in scope and cover issues outside the more complex laboratory accreditation schemes.

## Author Contributions

All authors listed have made a substantial, direct and intellectual contribution to the work, and approved it for publication.

### Conflict of Interest

The authors declare that the research was conducted in the absence of any commercial or financial relationships that could be construed as a potential conflict of interest.
